# A Convolutional Neural Network Based on Ultrasound Images of Primary Breast Masses: Prediction of Lymph-Node Metastasis in Collaboration With Classification of Benign and Malignant Tumors

**DOI:** 10.3389/fphys.2022.882648

**Published:** 2022-06-02

**Authors:** Chunxiao Li, Yuanfan Guo, Liqiong Jia, Minghua Yao, Sihui Shao, Jing Chen, Yi Xu, Rong Wu

**Affiliations:** ^1^ Department of Ultrasound, Shanghai General Hospital, Shanghai Jiao Tong University School of Medicine, Shanghai, China; ^2^ Shanghai Key Lab of Digital Media Processing and Transmission, Shanghai Jiao Tong University, Shanghai, China; ^3^ Department of Ultrasound, Zhongshan Hospital Wusong Branch, Fudan University, Shanghai, China

**Keywords:** breast neoplasms, ultrasonography, neural networks, lymph nodes, multi-task

## Abstract

**Purpose:** A convolutional neural network (CNN) can perform well in either of two independent tasks [classification and axillary lymph-node metastasis (ALNM) prediction] based on breast ultrasound (US) images. This study is aimed to investigate the feasibility of performing the two tasks simultaneously.

**Methods:** We developed a multi-task CNN model based on a self-built dataset containing 5911 breast US images from 2131 patients. A hierarchical loss (HL) function was designed to relate the two tasks. Sensitivity, specificity, accuracy, precision, F1-score, and analyses of receiver operating characteristic (ROC) curves and heatmaps were calculated. A radiomics model was built by the PyRadiomics package.

**Results:** The sensitivity, specificity and area under the ROC curve (AUC) of our CNN model for classification and ALNM tasks were 83.5%, 71.6%, 0.878 and 76.9%, 78.3%, 0.836, respectively. The inconsistency error of ALNM prediction corrected by HL function decreased from 7.5% to 4.2%. Predictive ability of the CNN model for ALNM burden (≥3 or ≥4) was 77.3%, 62.7%, and 0.752, and 66.6%, 76.8%, and 0.768, respectively, for sensitivity, specificity and AUC.

**Conclusion:** The proposed multi-task CNN model highlights its novelty in simultaneously distinguishing breast lesions and indicating nodal burden through US, which is valuable for “personalized” treatment.

## Introduction

The accurate diagnosis and precise staging of breast cancer (BC) is crucial for clinical decision-making, and is based on imaging examinations (Plevritis et al., 2018; Guo et al., 2018a; da Costa Vieira et al., 2017). According to the Tumor–Node–Metastasis (TNM) staging system, axillary lymph node (ALN) status is the principal factor for the clinical staging of BC (Giuliano et al., 2017). Ultrasound (US) is a routine imaging modality used to evaluate breast masses and is recommended for ALN assessment according to guidelines (Chang et al., 2020). Studies have shown the pooled sensitivity and specificity to be 0.87 and 0.80, respectively, and the area under the receiver operating characteristic curve (AUC) to be 0.9049, for conventional US in breast-lesion classification (Wubulihasimu et al., 2018). For ALNs, US can detect axillary lymph node metastases (ALNM) with sensitivity and specificity ranging from 26% to 76%, and 88% to 98%, respectively, based on morphologic criteria (Houssami et al., 2014). However, operator dependence and unclear diagnostic criteria for US of the breast limit its progress towards a precise diagnosis. These knowledge gaps call for confirmation of the presence/absence and extent of ALNM preoperatively for suspicious breast lesions concurrently for “tailored” treatment plans.

Several studies have shown that the US findings of primary BC might be associated with its malignancy and ALNM (Akissue de Camargo Teixeira et al., 2017; Bae et al., 2018; Guo et al., 2018b; Yu et al., 2018). Thus, one can predict malignancy and ALNM status simultaneously based on the morphologic characteristics of a primary breast mass. These relevant morphologic features include the tumor diameter, indistinct margins, and architectural distortion (Akissue de Camargo Teixeira et al., 2017; Bae et al., 2018; Guo et al., 2018b; Yu et al., 2018). However, some of these morphologic features are subjective and even invisible to the naked eyes of radiologists. Therefore, completing such sophisticated work is challenging for radiologists.

The advent of powerful artificial intelligence (AI) technology [particularly deep learning (DL) algorithms] could help to reduce the number of hospital visits and financial costs for patients because only US examination would be used (Sadoughi et al., 2018; Le et al., 2019). With self-learned features from US images of the breast, Zhao and others reported that the diagnostic performance of deep convolutional neural networks (CNNs) was comparable with that of expert radiologists, and also improved inter-observer agreement among radiologists (Zhao et al., 2019). Other studies have demonstrated the feasibility of predicting ALNM through the characteristics of US images of primary BC using DL models (Moon et al., 2018; Sun et al., 2020). However, AI studies have not explored the dual-task performance of breast-mass classification and ALNM prediction on US images.

We explored the feasibility of predicting both malignancy and ALNM using a unified CNN model based on US images of primary breast masses and exploring the underlying relationship between these two tasks. Such feasibility indicates that it is potential to build a multi-functional AI that could perform comprehensive analysis given US images. Specifically, the relationship among the multiple clinical tasks could be potentially learned as prior knowledge to guide feature optimization procedure and thus constrain the proposed CNN model to obtain a more accurate diagnosis result. Such a working mechanism could be considered as an extension of the diagnosis process of radiologist and could not be built by a radiomics model. Actually, it can be expected that we extend the model for more related AI functions by simply adding more task-specific heads, for example, molecular subtypes predictor.

## Materials and Methods

### Study Cohort

**FIGURE 1:**
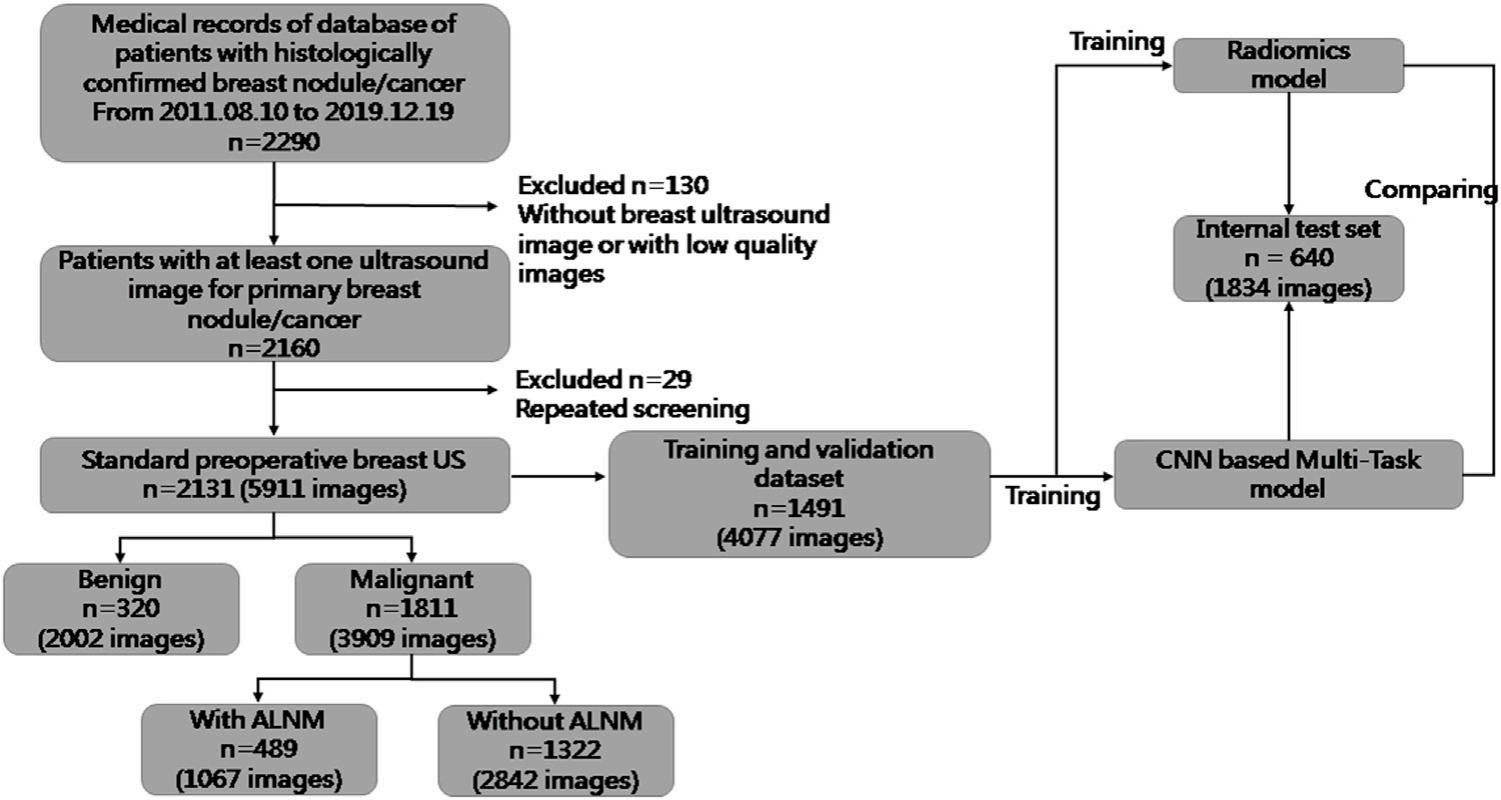
Flowchart of the procedures in the development and evaluation of our deep-learning model and radiomics model for malignancy prediction and ALNM prediction.

For this retrospective study, Institutional Review Board approval was obtained from our ethics committee (No. 2019KY055). The requirement for obtaining written informed consent from the subjects was waived by the Institutional Review Board. From August 2011 to December 2019, 5911 US images of breast masses were collected from 2131 patients (2120 females and 11 males). The nodules included in our study matched those detected by pathology after surgical resection. The inclusion criteria of this study were as follows: 1) All the breast masses were pathologically proven; 2) The patients had not undergone radiotherapy, chemotherapy and other anti-tumor treatments; 3) Breast cancer with ALNM were all ipsilateral with one malignant tumor. The flowchart of our study is shown as Figure 1. The dataset was split into sets (training, validation and testing), where images from the same patient were confirmed in the same part, and 30% of patients were chosen randomly once for all as the test set. All hyperparameters were chosen based on the performance on validation parts.

US images were acquired using US equipment obtained from Aixplorer (Super Imagine; Aix-en-Provence, France), Philips (IU22; Amsterdam, Netherlands), GE Healthcare (LOGIQ E9; Pittsburgh, PA, United States), Hitachi (EUB 8500; Tokyo, Japan), Esaote (MyLab™Twice; Genoa, Italy) and Siemens (Sequia512 and ACUSON S3000; Munich, Germany) with linear transducers of frequency 5–12 MHz.

### Data Preprocessing

An annotation tool for graphical images, LabelImg (https://github.com/tzutalin/labelImg), was used to crop the box-level region of interest (RoI), which was delineated manually and confirmed by two radiologists with >5 years of experience in breast US interpretation. Two radiologists delineated different parts of the study cohort and therefore there is no discrepancy. To achieve data diversity and to prevent overfitting, strong data augmentation was adopted for image processing. Geometric transformations of random images (including random cropping and scaling, random flipping, and random color distortion) were used. The augmented images were resized to 256 × 256 pixels before being fed into the model. We implemented the training-and-testing framework with PyTorch (https://pytorch.org/).

### Hierarchical Loss for Prediction Consistency Between Two Tasks

We proposed a multi-task framework to predict the malignancy of the tumor and ALNM simultaneously. Tumors with ALNM are always malignant. Accordingly, HL was designed to constrain the model to achieve consistent predictions between two tasks (if there was a high/low score for ALNM, there should also be a high/low score for malignancy) and formed as:

$$L_{IH}=\frac{1}{N}\overset{N}{\underset{i=1}{\sum }}\max\left(0,\;\hat{y_{i2}}-\hat{y_{i1}}-m\right),$$

where N is the batch size and $\hat{y_{i2}}$ is the model prediction of ALNM for sample i . Correspondingly, $\hat{y_{i1}}$ is the model prediction of malignancy for sample i . Parameter m is the margin. HL produces gradients only if the relative prediction confidence between two tasks is larger than the margin. A large margin denotes a “looser” constraint, which allows a larger gap of prediction confidence between two tasks. A small margin indicates a more stringent constraint. The margin is set as 0.1 for all experiments as selected based on the preliminary experiments conducted on training and validation cohort. To be specific, we performed a grid search to find the margin that resulted in best classification performance on validation cohort.

### Multi-Task Convolutional Neural Network Framework

**FIGURE 2:**
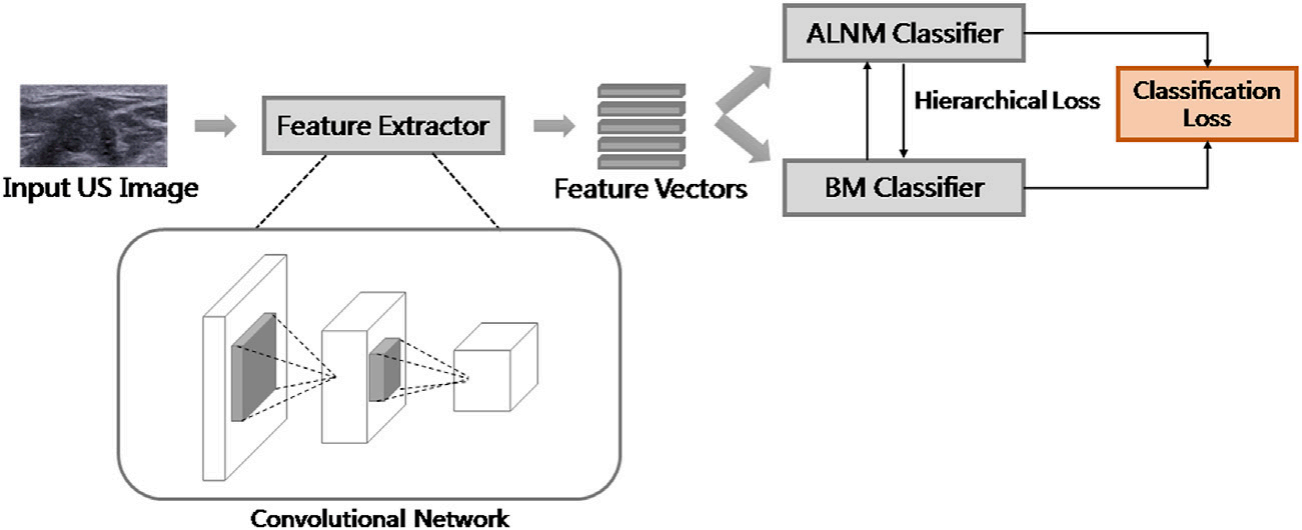
Overall framework of our model.

The overall learning architecture of our model is illustrated in Figure 2. We used ResNet as feature extractor. The classifiers were fully connected layers with a sigmoid function as the activation. The sigmoid function scaled the output of classifiers to a range of [0,1], which could be explained as the probability of a malignant tumor with ALNM. Binary cross entropy (BCE) loss was used for classification loss because both tasks could be formulated as a binary classification. The overall loss function for training was the weighted sum of BCE loss and HL, as follows:

$$L_{all}=\lambda L_{IH}+L_{BCE},$$

where the hyperparameter λ was set as 0.05 by default as selected on the validation set.

### Radiomics Model

We also built an advanced radiomics model proposed by Sun et al. (2020) as a baseline to compare with our proposed multitask CNN. The radiomics model extracts statistical features from ultrasound images and performs classification with random forest algorithms. The advantages of our proposed multitask CNN framework against the radiomics model are twofold: 1) The features extracted by CNN model are optimized by the classification objective, while those extracted by the radiomics model are manually designed, which are difficult to be generalized for extensively varied image contents and inclined to be non-optimal towards classification; 2) In our framework, the two tasks could be performed simultaneously without re-training and more importantly, the relationship between the two tasks could be utilized to enhance the feature representation and results in classification performance improvements. For each US image, 102 radiomics features were extracted using Pyradiomics (https://pyradiomics.readthedocs.io) (Supplementary Table S1), including intensity features and texture features. When extracting these features, data augmentation is not utilized for the reproduction of extracted features. Shape features were not considered because pixel-level labeling of RoIs required very expensive manual labor. The Boruta feature-selection algorithm was used for dimension reduction to avoid overfitting. The selected features for each task are summarized in Supplementary Table S2. We used the Python package Boruta for implementation (https://pypi.org/project/Boruta/). The classifier of our radiomics model was the Random Forest (RF) classifier with a maximum depth of 300. The scikit-learn package (https://scikit-learn.org/stable/) in Python was used for implementation.

### Evaluation and Statistical Analyses

The difference in the characteristics of patients and lesions were assessed with the chi-square test or Student’s *t*-test, where appropriate. Descriptive data are the mean ± SD. Statistical tests were two-sided and undertaken with the scipy package (https://www.scipy.org/) in Python. *p* < 0.05 was considered significant. Experiments were repeated five times to ensure reproducibility. We introduced gradient-weighted class activation mapping (Grad-CAM) (Selvaraju et al., 2017) to visualize the feature extracted by the model (heatmap). Accuracy, sensitivity, specificity, F1-score and precision were used for performance evaluation. It should be noted that these metrics are calculated according to the relationship between model prediction and the ground truth. Actually, these metrics are calculated according to the number of true-positive, true-negative, false-positive and false-negatives, the definition of which regarding the pathological result could be found in Appendix. It should be also noted that calculating these metrics require a discrete prediction while the output of model is a real value in range [0, 1] interpreted as the probability of being positive. Therefore, we follow the common practice of setting a threshold as 0.5 on both ALNM task and BM task. That is, if the output of model is greater than 0.5, the prediction would be considered as positive. To further evaluate the performance of model under different thresholds, receiver operating characteristic (ROC) curve would be plotted and the area under curve (AUC) would be reported.

### Experimental Configuration

ResNet50 (He et al., 2016) was used for all feature extractors in our DL models. The Adam optimization algorithm was selected to train our models with initial learning rate of 1e-4. All models were trained for 200 epochs, with the learning rate reduced by a factor of 10 every 50 epochs. Two ablation experiments were conducted whereby the two tasks were undertaken independently and then together but without the HL function. In the present study, “Single-BM” denotes the algorithm using ResNet to conduct malignancy prediction, “Single-ALNM” denotes the algorithm using ResNet to conduct ALNM prediction, and “Multi-task” denotes the algorithm which uses ResNet as the shared backbone and two classifiers to predict the scores of the two tasks simultaneously without the HL term.

## Results

### Patient Data

The clinical and pathological results of the whole dataset are shown in Table 1. Finally, 1491 patients (mean age± SD: 54.25 ± 14.29 years; range: 17–88 years) with 4077 images were used for the training set and validation set, and 640 patients (mean age± SD: 55.04 ± 14.49 years; range: 21–91 years) with 1834 images were used for the test set. For prediction of breast-mass malignancy, the dataset comprised 3909 images of 1811 malignant breast lesions and 2002 images of 320 benign lesions. For ALNM prediction, 489 ALN-positive patients with 1067 images and 1322 ALN-negative patients with 2842 images were sorted for investigation.

**TABLE 1:** Characteristics of patients and lesions.

Characteristic	Training and Validation	Testing	Total	*p*
Whole Datasets				
Number of patients	1491	640	2131	
Number of images	4077	1834	5911	
Age (mean ± SD)	54.25 ± 14.29	55.04 ± 14.49	54.49 ± 14.35	0.25
Sex				0.90
Female	1480	640	2120	
Male	8	3	11	
Tumor diameter (cm)				0.99
≤ 2.0	925	396	1321	
2.0–5.0	538	230	768	
≥5.0	29	13	42	
Histological type				
Malignant				0.95
Invasive ductal carcinoma	942	398	1340	
Invasive lobular carcinoma	22	11	33	
Ductal carcinoma *in situ*	110	49	159	
Others	193	86	279	
Benign				0.12
Adenopathy	63	20	83	
Fibroadenoma	40	17	57	
Intraductal papillary carcinoma	4	6	10	
Others	117	53	170	
Surgery type for Breast cancer				0.26
Modified radical mastectomy	482	208	690	
Mastectomy	156	51	207	
Lumpectomy	486	212	698	
Radical mastectomy	48	20	68	
Unsure	95	53	148	
Location				0.65
Right	734	346	1080	
Left	725	326	1051	
Family history				0.99
Yes	54	24	78	
No	1437	616	2053	
Malignancy prediction				0.96/0.2
Number of malignant tumors (patients/images)	1267/2674	544/1235	1811/3909	
Number of benign masses (patients/images)	224/1403	96/599	320/2002	
ALNM prediction				0.61/0.72
No lymph-node metastasis	920/1939	402/903	1322/2842	
Lymph-node metastasis (patients/images)	347/735	142/332	489/1067	
ALNM ≥ 2	242/510	95/219	337/729	
ALNM ≥ 3	179/389	68/147	247/536	
ALNM ≥ 4	122/259	52/111	174/370	

ALNM, axillary lymph-node metastasis.

### Convolutional Neural Network Model Versus Radiomics Model

**FIGURE 3:**
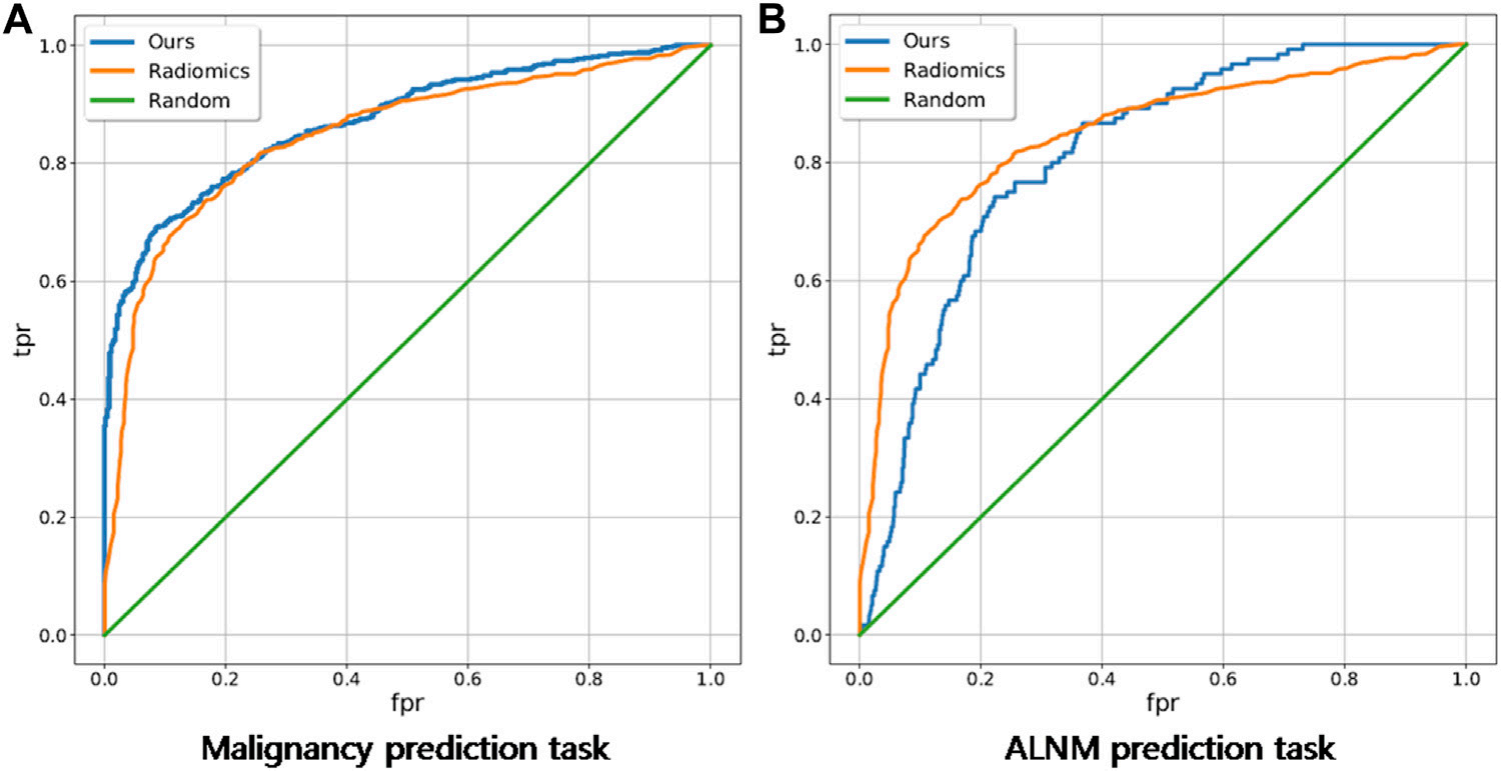
Receiver operating characteristic curves of our deep-learning model and a traditional radiomics model on malignancy prediction and ALNM prediction. **(A)** and ALNM prediction **(B)** tasks.

For the BM task, our model showed an encouraging improvement in performance, with an AUC of 0.878, sensitivity of 83.5% and specificity of 71.6% compared with those in the radiomics model, which achieved an AUC of 0.848 (*p* < 0.001) (Table 2; Figure 3). With respect to ALNM prediction, the AUC, sensitivity, and specificity of our model was 0.836, 76.9% and 78.3%, respectively, which were all superior to those of the radiomics model (*p* < 0.05 for all metrics).

**TABLE 2:** Performance of the deep-learning model and radiomics model on the test cohort on ALNM prediction and benign/malignant classification task.

	ALNM					
	ACC	SE	Prec	SP	F1	AUC
Radiomics	0.751	0.700	0.220	0.757	0.335	0.804
Our method	0.782 ± 0.01 (0.753, 0.808)	0.769 ± 0.025 (0.699, 0.838)	0.259 ± 0.008 (0.236, 0.281)	0.783 ± 0.013 (0.747, 0.819)	0.387 ± 0.009 (0.362, 0.412)	0.836 ± 0.011 (0.805, 0.866)
*p*	0.0001	0.0002	<0.0001	0.002	<0.0001	0.0002
**BM**						
Radiomics	0.780	0.801	0.802	0.754	0.801	0.848
Our method	0.782 ± 0.01 (0.754, 0.809)	0.835 ± 0.007 (0.815, 0.854)	0.784 ± 0.012 (0.750, 0.817)	0.716 ± 0.019 (0.663, 0.768)	0.810 ± 0.008 (0.787, 0.832)	0.878 ± 0.007 (0.858, 0.897)
*p*	0.666	<0.0001	0.01	0.002	0.04	<0.001

ACC, accuracy; SE, sensitivity, Prec, precision, SP, specificity; AUC, area under the ROC, curve; ALNM, axillary lymph node metastasis; BM, Benign/malignant classification. When applicable, statistical quantifications are demonstrated with 95% confidential interval (CI).

### Evaluation of Hierarchical Loss Function

**FIGURE 4:**
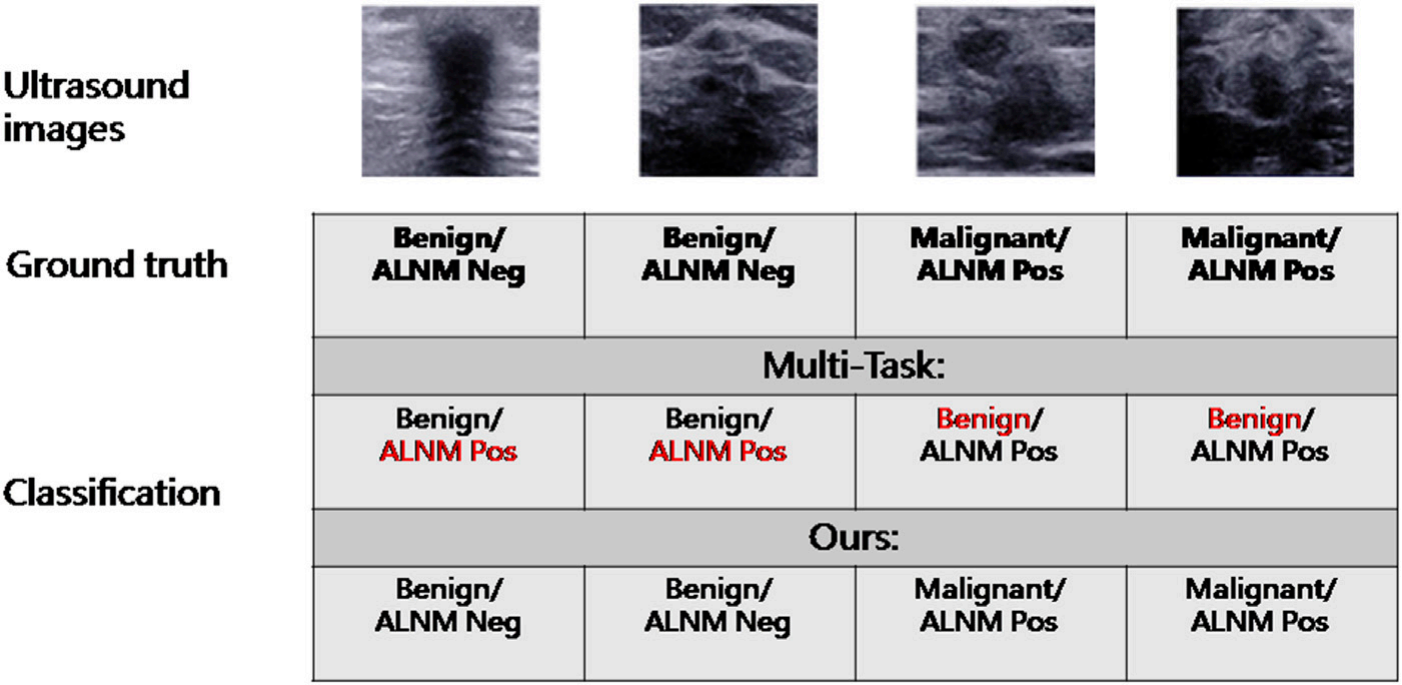
Example of reduced inconsistency by introduction of a hierarchical-loss function.

The malignancy prediction for Multi-Task showed no significant difference from Single-BM (0.874 *vs*. 0.871, *p* = 0.379) (Table 3). The ALNM prediction for Multi-Task showed no significant difference from Single-BM (0.820 *vs*. 0.817, *p* = 0.597), and we noticed that combining the two tasks together did not necessarily improve the performance because the collaboration between the two tasks was not well-built. The AUC value of ALNM prediction with HL introduction surpassed that of Multi-Task (0.836 *vs*. 0.817, *p* = 0.009), which indicated that the prior knowledge that tumors with ALNM are always malignant imposed a valid interactive constraint between two classifiers. All *p*-values are listed in Supplementary Table S3. We further study the impact of HL for reducing inconsistent predictions. The percentages of two types of inconsistency error in the test set for models without HL and with HL are 7.5% vs. 4.2% and 2.0% vs. 1.1%, respectively. The impact of HL for reducing inconsistent predictions are shown in Figure 4.

**TABLE 3:** Ablation studies for our proposed model.

	ALNM					
Methods	ACC	SE	Prec	SP	F1	AUC
Single-BM	**\**	**\**	**\**	**\**	**\**	**\**
Single-ALNM	0.774 ± 0.01 (0.746, 0.801)	0.724 ± 0.05 (0.585, 0.862)	0.243 ± 0.006 (0.226, 0.259)	0.778 ± 0.015 (0.736, 0.819)	0.365 ± 0.01 (0.337, 0.392)	0.820 ± 0.010 (0.792, 0.847)
Multi-Task	0.771 ± 0.008 (0.748, 0.793)	0.673 ± 0.018 (0.623, 0.723)	0.232 ± 0.006 (0.215, 0.248)	0.780 ± 0.01 (0.752, 0.807)	0.345 ± 0.008 (0.322, 0.367)	0.817 ± 0.007 (0.797, 0.836)
Ours	0.782 ± 0.01 (0.753, 0.808)	0.769 ± 0.025 (0.699, 0.838)	0.259 ± 0.008 (0.236, 0.281)	0.783 ± 0.013 (0.747, 0.819)	0.387 ± 0.009 (0.362, 0.412)	0.836 ± 0.011 (0.805, 0.866)
**BM**						
Single-BM	0.770 ± 0.005 (0.756, 0.783)	0.826 ± 0.01 (0.798, 0.853)	0.774 ± 0.007 (0.754, 0.793)	0.703 ± 0.013 (0.666, 0.739)	0.799 ± 0.005 (0.785, 0.812)	0.871 ± 0.006 (0.854, 0.887)
Single-ALNM	**\**	**\**	**\**	**\**	**\**	**\**
Multi-Task	0.773 ± 0.005 (0.759, 0.786)	0.830 ± 0.023 (0.766, 0.893)	0.776 ± 0.016 (0.731, 0.820)	0.702 ± 0.034 (0.607, 0.796)	0.801 ± 0.004 (0.789, 0.812)	0.874 ± 0.004 (0.862, 0.885)
Ours	0.782 ± 0.01 (0.754, 0.809)	0.835 ± 0.007 (0.815, 0.854)	0.784 ± 0.012 (0.750, 0.817)	0.716 ± 0.019 (0.663, 0.768)	0.810 ± 0.008 (0.787, 0.832)	0.878 ± 0.007 (0.858, 0.897)

Single-BM, training on malignancy prediction task only; Single-ALNM, training on ALNM prediction task only; Multi-task, combination of two tasks together without hierarchical loss. When applicable, statistical quantifications are demonstrated with 95% confidential interval (CI).

### Deep Learning Interpretability With Gradient-Weighted Class Activation Mapping

**FIGURE 5:**
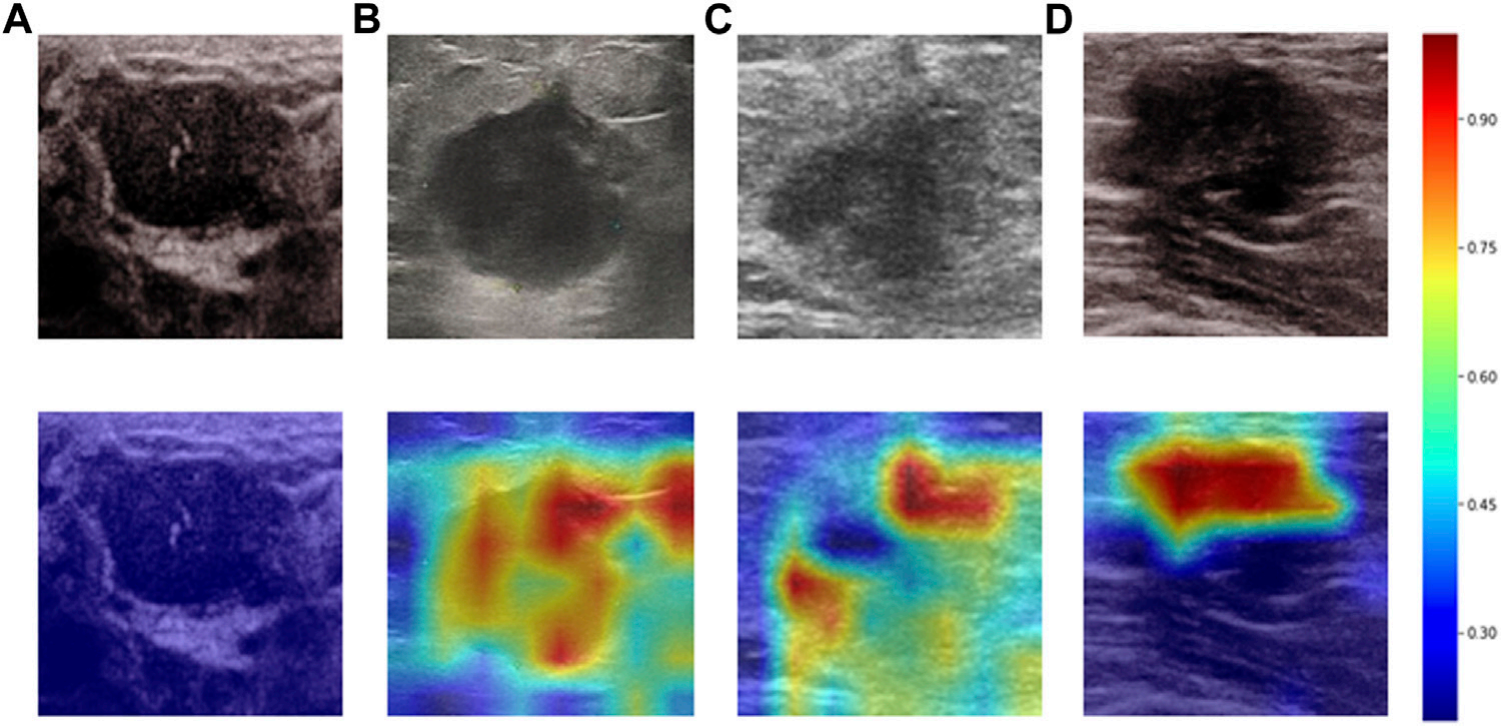
Heatmaps generated by Grad-CAM. The color ranges from blue to red, and represents the significance value of the region ranging from low to high, respectively, for the prediction of a malignant tumor or ALNM. **(A)** Benign case which was classified correctly. **(B)** Malignant masses without ALNM. **(C)** and **(D)** Malignant masses.

**FIGURE 6:**
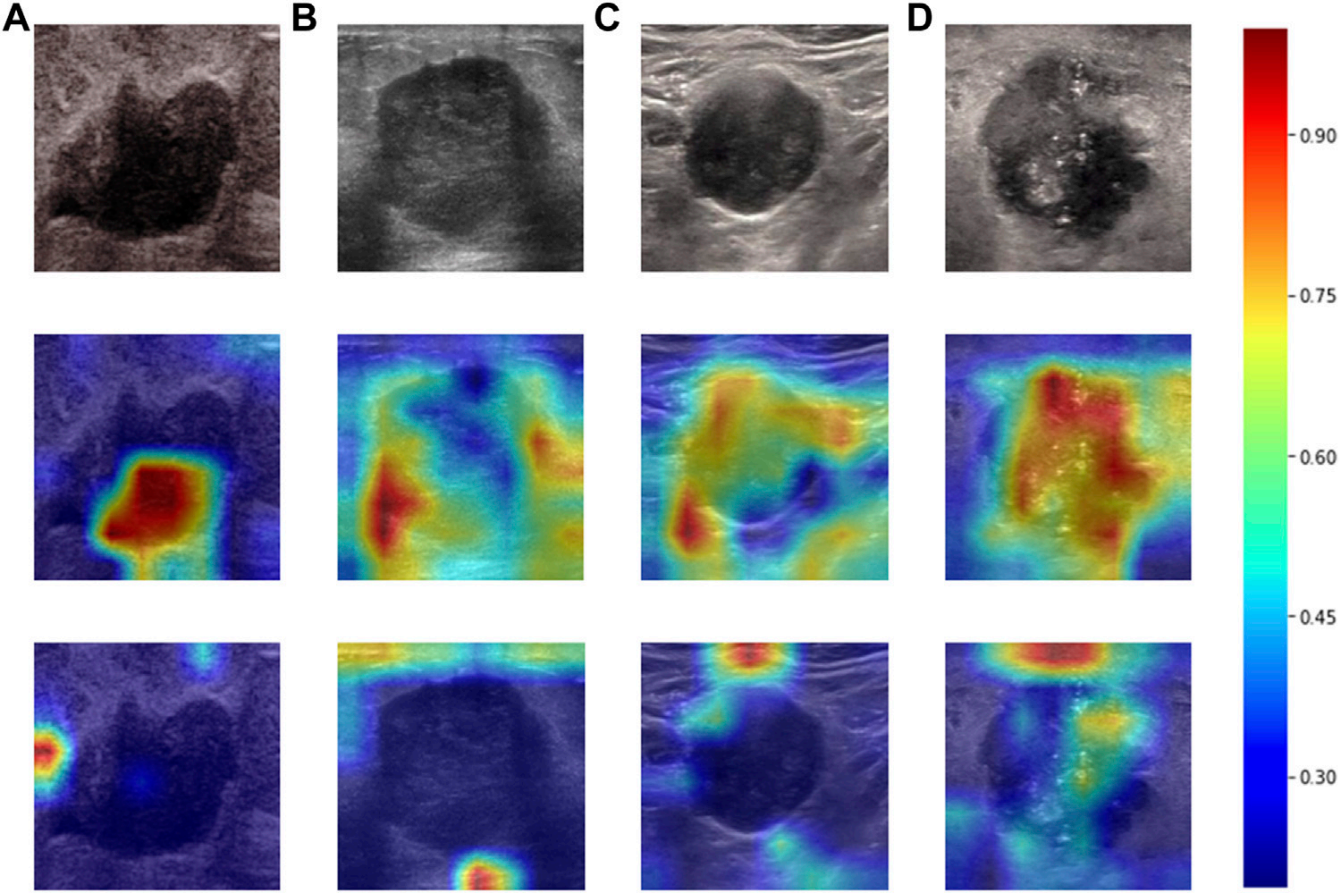
Heatmaps produced using Grad-CAM. The first row **(A–D)** shows US images from patients with ALNM. The second row shows heatmaps generated from our model that performed ALNM prediction and malignancy prediction. The third row shows heatmaps generated from the model performing only ALNM prediction.

Heatmaps are shown as Figure 5. The color change from blue to red represents the significance of the regions for prediction of a malignant tumor or ALNM ranging from low to high, respectively. In Figures 5B,C, intratumoral regions and peritumoral regions are highlighted with hot colors, and contribute dominantly to the prediction of malignant tumors or ALNM. We also studied the average CAM response over different distances to image centers on the test set (Supplementary Figure S1). The result indicated that pixels closer to the image center (which denotes a higher probability of being the inratumoral region) contributed more to the prediction. Pixels distant from the image center tended to be the intratumoral region and background. In Figure 6, the heatmap of the model supervised only by the ALNM label (third row) showed that the model highlighted only the smooth peritumoral region rather than considering the peritumoral and intratumoral information together. However, for the model supervised by malignancy and ALNM labels (second row), the peritumoral and intratumoral regions were highlighted. These data might explain the reason for the performance gain of ALNM prediction: introduction of HL and malignancy supervision.

### Prediction of the Nodal Burden of Axillary Lymph-Node Metastasis

To calculate the nodal burden of ALNM, we built several binary classification tasks whereby positive samples were images from patients with ALNM number no less than a given number ranging from 1 to 4. The detailed performance evaluation of the CNN method and radiomics method on this set of binary classification tasks is summarized in Table 4. The score for accuracy, sensitivity, specificity, and the AUC for the number of ALNM ≥ 2, ALNM ≥ 3 and ALNM ≥ 4 by the CNN model was, respectively: 63.4%, 67.2%, 63.0%, and 0.715; 63.6%, 77.3%, 62.7%, and 0.752; 75.3%,66.6%, 76.8%, and 0.768.

**TABLE 4:** Performance of the radiomics model and our model on prediction of the exact number of lymph-node metastases.

Task	Model	Metrics					
		ACC	SE	Prec	SP	F1	AUC
ALNM ≥ 2	Radiomics	0.611	0.592	0.141	0.613	0.228	0.667
	Ours	0.634 ± 0.023 (0.570, 0.697)	0.672 ± 0.039 (0.563, 0.780)	0.163 ± 0.005 (0.149, 0.176)	0.63 ± 0.291 (0.549, 0.710)	0.263 ± 0.006 (0.246, 0.279)	0.715 ± 0.015 (0.673, 0.756)
ALNM ≥ 3	Radiomics	0.575	0.607	0.087	0.573	0.152	0.602
	Ours	0.636 ± 0.016 (0.591, 0.680)	0.773 ± 0.051 (0.631, 0.914)	0.121 ± 0.002 (0.115, 0.126)	0.627 ± 0.002 (0.621, 0.632)	0.211 ± 0.004 (0.199, 0.222)	0.752 ± 0.013 (0.715, 0.788)
ALNM ≥ 4	Radiomics	0.683	0.417	0.06	0.695	0.105	0.588
	Ours	0.753 ± 0.018 (0.703, 0.802)	0.666 ± 0.065 (0.485, 0.846)	0.119 ± 0.006 (0.102, 0.135)	0.768 ± 0.012 (0.734, 0.801)	0.202 ± 0.013 (0.165, 0.238)	0.768 ± 0.009 (0.743, 0.792)

ACC, accuracy; SE, sensitivity, Prec, precision, SP, specificity; AUC, area under the ROC curve. When applicable, statistical quantifications are demonstrated with 95% confidential interval (CI).

## Discussion

We successfully developed a multi-task architecture to integrate two traditional single-mode classification tasks to achieve prediction of ALNM and malignancy in parallel. In the practical work, the radiologist must make the classification of breast nodules firstly, and then the lymph node status assessment. This sequence is a comprehensive diagnosis. However, many previous research for the prediction of lymph node were based on selective breast cancer tumors, do not tally with the actual diagnosis process. Therefore, in our opinion, this study is consistent with the clinical diagnostic process and has good clinical relevance based on clinical issues. The HL function was utilized to embed the prior constraint between the two tasks that tumors predicted with ALNM should obtain a label of “malignancy” while there must be no metastasis to LNs in a benign breast tumor. Ablation studies were undertaken to evaluate the impact of the HL function, which was shown to be effective for reducing inconsistent prediction errors. This is an innovative study that is different from the previous task of single classification or lymph node prediction model. In clinical practice, if US examination cannot provide a correct assessment of lymph node status, patients may need MRI examination or sentinel lymph node biopsy to confirm the status of lymph nodes. Using our model, radiologists and physicians could reach a diagnosis quickly without resorting to other examinations efficiently and economically.

Numerous studies suggested that DL provides a powerful assistant tool for radiologists to reduce their workload (Xiao et al., 2018; Wu et al., 2019). The excellent ability of CNNs to extract image features was proved to be superior to radiomics using CT, US or MRI (Truhn et al., 2019; Caballo et al., 2020; Sun et al., 2020). Consistent with those results, our CNN model was also superior to the radiomics model based on the same dataset. Han and others used a CNN framework based on large-scale data to differentiate breast lesions on US images, and achieved an accuracy of ∼0.9, sensitivity of ∼0.86, and specificity of ∼0.96 (Han et al., 2017). Our model achieved a relatively lower specificity (0.72) due to the sample composition, in which the number of breast tumors was in the majority. As a retrospective study, images from multiple machines were also responsible for this classification performance. However, our model had the possibility of eliciting reproducibility and repeatability in daily practice. Moreover, based on this preliminary study, our next work is to build a comprehensive model by combining clinical data and multimodal ultrasound image data to accomplish this dual task, which may yield better diagnostic performance.

Most importantly, our study provides a valid approach to predict the nodal burden of ALNM of malignant breast tumors. Zhou and others revealed that the best-performing model significantly outperformed the three experienced radiologists in ALNM prediction, but detailed data were not provided (Zhou et al., 2020). The nodal burden of ALNM is an important parameter for clinical treatment. According to the results of the Z0011 trial from the American College of Surgeons Oncology Group, the clinical treatment for ALN has been changed further worldwide. This is because dissection of ALNs does not affect the overall survival or disease-free survival of patients with T1–T2 BC, or <3 positive sentinel LNs under different treatment regimens (Giuliano et al., 2016). In the TNM staging system, ALNM ≥ 4 increases the clinical stage of BC, which corresponds to a change in the treatment regimen (Giuliano et al., 2017). Compared with the study conducted by Zheng et al. (2020), in addition to prediction of ALN status of N0, N+(≥1),N+(1–2), N+(≥3), our study carried out more attempts at lymph node metastasis status (N ≥ 4), which is important reference information for the clinical staging of breast cancer.

Our study showed moderate performance in prediction of ALNM number, which might have been due to our small sample size. Nevertheless, we demonstrated the potential of CNN model in predicting the ALN staging of BC. Our model achieved better performance on ALNM prediction with higher nodal burden, which indicates that there were more hard samples among breast tumors with a lower nodal burden. For training the CNN model, these hard samples might provide incorrect gradients, which should be ignored to prevent the model from overfitting. A possible explanation for this scenario could be that the metastatic biological behaviors of breast tumors with a lower nodal burden are inconspicuous compared with those of a heavier breast tumor which displays well-marked morphologic features (Bae et al., 2018; Yu et al., 2018). Moreover, our results are in accordance with the fact that US has greater accuracy in detecting a heavy nodal burden of BC compared with lower nodal involvement (Rukanskienė et al., 2020).

To assist radiologists in understanding our CNN model and reveal the inter relationship of the two tasks, we visualized the heatmap (which shows the important parts of the US image of the breast during prediction). We discovered that peritumoral and intratumoral regions contributed to prediction of ALNM. This result is in accordance with studies suggesting that the biological changes of peritumoral regions might lead to metastatic spread (Wu et al., 2017; Cheon et al., 2018). This result might also explain the reason for the performance gain of ALNM prediction by introducing HL and malignancy supervision. The useful interaction between two tasks provides extra information, thereby forcing model to consider intratumoral and peritumoral regions to make more precise predictions.

Our study had two main limitations. First, the self-built dataset was of limited size. Further expansion of data with larger and multicenter datasets should be undertaken in the future. Second, our data were only derived from two-dimensional US images, future studies which focus on a combination of multi-mode US images of breast masses should be taken to improve the diagnostic performance of ALN status.

## Conclusion

We developed a novel multi-task CNN model to predict malignancy and detailed LN status simultaneously based on US images of primary BC. For patients, US can be a cost-effective, convenient, and functional alternative examination. For physicians, preoperative evaluation could be accomplished with fewer screening items and shorter intervals between visits. Our CNN model has considerable potential for assisting clinically precise treatment for BC.

## Data Availability

The datasets presented in this article are not readily available because This data involves patient privacy information that we have chosen not to disclose. Requests to access the datasets should be directed to wurong7111@163.com.

## Abbreviations

CNN, Convolutional neural network; ROC, Receiver operating characteristic; AUC, Area under the ROC curve; TNM, Tumor, Node, Metastasis; AI, Artificial intelligence; DL, Deep learning; ROI, Region of interest; SD, Standard deviation.
