# Supplementary material for: A Convolutional Neural Network Based on Ultrasound Images of Primary Breast Masses: Prediction of Lymph-Node Metastasis in Collaboration With Classification of Benign and Malignant Tumors
Source: Front Physiol. 2022 Jun 2;13:882648. doi: 10.3389/fphys.2022.882648 (PMC9205241; doi:10.3389/fphys.2022.882648)
Supplement: Supplementary file 1 [file DataSheet1.docx]

**Appendix**

**Summary:**

The appendix includes 3 tables and 2 figures. Table S1 and S2 demonstrate the selected radiomics features. Table S3 shows the full P-value of methods listed in Table 3. Figure S1 and S2 illustrate the average Grad-CAM response v.s. distance to image center and response for failed cases.

**Definition of evaluation metrics**

The classification metrics accuracy (ACC), sensitivity (SE), specificity (SP), precision (Prec), f1-score (F1) are formalized as follows:

$$ACC=\frac{TP+TN}{TP+FP+TN+FN}$$

$$SE=\frac{TP}{TP+FN}$$

$$SP=\frac{TN}{TN+FP}$$

$$Prec=\frac{TP}{TP+FP}$$

$$F1=2\times\frac{Prec\times SE}{Prec+SE}$$

TPs, FPs, TNs and FNs are defined according to the model output and pathological examination. Specifically, for benign/malignant classification task for example, the output of model would be a number in range [0, 1] representing the probability of being malignant. Then, if the output is greater than 0.5, it would be classified into malignant. The definition of TPs, FPs, TNs and FNs are then as follows:

• TP: The ground truth is malignant and prediction is malignant.

• FP: The ground truth is benign but the prediction is malignant.

• TN: The ground truth is benign and the prediction is benign.

• FN: The ground truth is malignant but the prediction is benign.

For ALNM task, the definition is similar according to the result of pathological examination.

**Extracted radiomics features**

Table S1

A summary of the radiomics features extracted. Informational Measure of Correlation have two calculation methods, respectively, which can be found in the study by Aerts H.J. et al [1].

| **Feature Classes** | | **Feature Names** |
| --- | --- | --- |
| Shape Features | | Volume, Elongation, Surface Area, Max 2D Diameter, Mesh Volume,Major Axis Length, Max 2D Diameter Row,Max 2D Diameter Column, Surface Volume Ratio, Sphericity, Minor Axis Length |
| Intensity Features | | Maximum, Median, Minimum, Mean Energy, Entropy, Variance, Kurtosis, Root Mean Square, Skewness, 10th Percentile, 90th Percentile, Mean Absolute Deviation, Uniformity, Range, Robust Mean Absolute Deviation, Total Energy, Interquartile Range |
| Texture  Features | GLCM  Features | Contrast, Correlation, Autocorrelation, Cluster Tendency, Sum Average, Sum Entropy, Sum Squares, Difference Average, Difference Variance, Difference Entropy, Cluster Prominence, Cluster Shade, Maximum Probability, Inverse Difference Moment, Informational Measure of Correlation 1/2, Inverse Difference Moment Normalized, Inverse Difference Normalized, Inverse Difference, Inverse Variance, Maximal Correlation Coefficient, Joint Average, Joint Energy, Joint Entropy |
|  | GLDM  Features | Dependence Entropy, Dependence Non-Uniformity, Dependence Non-Uniformity Normalized, Dependence Variance, Gray-Level Non-Uniformity, Gray-Level Variance, High Gray-Level Emphasis, Large Dependence Emphasis, Large Dependence High Gray-Level Emphasis, Large Dependence Low Gray-Level Emphasis, Low Gray-Level Emphasis, Small Dependence Emphasis, Small Dependence High Gray-Level Emphasis, Small Dependence Low Gray-Level Emphasis |
|  | GLRLM  Features | Gray-Level Non-uniformity, Gray-Level Non-uniformity Normalized, Gray-Level Variance, High Gray-Level Run Emphasis, Long Run Emphasis, Long Run High Gray-Level Emphasis, Long Run Low Gray-Level Emphasis, Low Gray-Level Run Emphasis, Run Entropy, Run Length Non-Uniformity, Run Length Non-Uniformity Normalized, Run Percentage, Run Variance, Short Run Emphasis, Short Run High Gray-Level Emphasis, Short Run Low Gray-Level Emphasis |
|  | GLSZM  Features | Gray-Level Non-Uniformity, Gray-Level Non-Uniformity Normalized, Gray-Level Non-Uniformity Normalized, High Gray-Level Zone Emphasis, Large Area Emphasis, Large Area High Gray-Level Emphasis, Large Area Low Gray-Level Emphasis, Low Gray-Level Zone Emphasis, Size Zone Non-Uniformity, Size Zone Non-Uniformity Normalized, Small Area Emphasis, Small Area High Gray-Level Emphasis, Small Area Low Gray-Level Emphasis, Zone Entropy, Zone Percentage, Zone Variance |
|  | NGTDM  Features | Coarseness, Contrast, Busyness, Complexity, Strength |

Table S2

1. The selected features by Borutal Algorithm for building our radiomics models for Malignancy Prediction task. We perform t-test between training and testing features and Wilcoxon rank-sum test to assess the univariant association between the selected imaging features and the Malignancy.

| No. | Selected Features | Type | Impt | P(t) | P(RS) |
| --- | --- | --- | --- | --- | --- |
| 0 | 90Percentile | Intensity | 0.006 | 0.013 | 0.000 |
| 1 | Energy | Intensity | 0.009 | 0.000 | 0.000 |
| 2 | Entropy | Intensity | 0.006 | 0.009 | 0.000 |
| 3 | InterquartileRange | Intensity | 0.007 | 0.000 | 0.000 |
| 4 | Kurtosis | Intensity | 0.010 | 0.002 | 0.000 |
| 5 | Maximum | Intensity | 0.006 | 0.328 | 0.000 |
| 6 | MeanAbsoluteDeviation | Intensity | 0.015 | 0.000 | 0.000 |
| 7 | Median | Intensity | 0.006 | 0.397 | 0.000 |
| 8 | Minimum | Intensity | 0.016 | 0.820 | 0.000 |
| 9 | RobustMeanAbsoluteDeviation | Intensity | 0.011 | 0.000 | 0.000 |
| 10 | Skewness | Intensity | 0.011 | 0.212 | 0.000 |
| 11 | TotalEnergy | Intensity | 0.008 | 0.000 | 0.000 |
| 12 | Uniformity | Intensity | 0.007 | 0.035 | 0.000 |
| 13 | Variance | Intensity | 0.013 | 0.000 | 0.000 |
| 14 | Autocorrelation | GLCM-Texture | 0.007 | 0.133 | 0.000 |
| 15 | ClusterProminence | GLCM-Texture | 0.012 | 0.000 | 0.000 |
| 16 | ClusterShade | GLCM-Texture | 0.011 | 0.302 | 0.000 |
| 17 | ClusterTendency | GLCM-Texture | 0.010 | 0.000 | 0.000 |
| 18 | Contrast | GLCM-Texture | 0.011 | 0.001 | 0.000 |
| 19 | Correlation | GLCM-Texture | 0.010 | 0.212 | 0.000 |
| 20 | DifferenceAverage | GLCM-Texture | 0.006 | 0.029 | 0.000 |
| 21 | DifferenceEntropy | GLCM-Texture | 0.006 | 0.056 | 0.000 |
| 22 | DifferenceVariance | GLCM-Texture | 0.015 | 0.001 | 0.000 |
| 23 | Idmn | GLCM-Texture | 0.005 | 0.063 | 0.000 |
| 24 | Idn | GLCM-Texture | 0.006 | 0.104 | 0.000 |
| 25 | Imc1 | GLCM-Texture | 0.008 | 0.822 | 0.000 |
| 26 | Imc2 | GLCM-Texture | 0.007 | 0.118 | 0.000 |
| 27 | JointAverage | GLCM-Texture | 0.006 | 0.547 | 0.000 |
| 28 | JointEnergy | GLCM-Texture | 0.006 | 0.133 | 0.000 |
| 29 | JointEntropy | GLCM-Texture | 0.006 | 0.013 | 0.000 |
| 30 | MCC | GLCM-Texture | 0.014 | 0.616 | 0.000 |
| 31 | MaximumProbability | GLCM-Texture | 0.008 | 0.391 | 0.000 |
| 32 | SumAverage | GLCM-Texture | 0.007 | 0.547 | 0.000 |
| 33 | SumEntropy | GLCM-Texture | 0.007 | 0.020 | 0.000 |
| 34 | SumSquares | GLCM-Texture | 0.011 | 0.000 | 0.000 |
| 35 | DependenceEntropy | GLDM-Texture | 0.007 | 0.024 | 0.000 |
| 36 | DependenceNonUniformity | GLDM-Texture | 0.012 | 0.046 | 0.000 |
| 37 | DependenceVariance | GLDM-Texture | 0.012 | 0.029 | 0.000 |
| 38 | GrayLevelNonUniformity | GLDM-Texture | 0.010 | 0.224 | 0.000 |
| 39 | GrayLevelVariance | GLDM-Texture | 0.011 | 0.000 | 0.000 |
| 40 | HighGrayLevelEmphasis | GLDM-Texture | 0.006 | 0.127 | 0.000 |
| 41 | LargeDependenceHighGrayLevelEmphasis | GLDM-Texture | 0.006 | 0.320 | 0.000 |
| 42 | LargeDependenceLowGrayLevelEmphasis | GLDM-Texture | 0.007 | 0.053 | 0.000 |
| 43 | LowGrayLevelEmphasis | GLDM-Texture | 0.006 | 0.057 | 0.000 |
| 44 | SmallDependenceEmphasis | GLDM-Texture | 0.007 | 0.007 | 0.000 |
| 45 | SmallDependenceHighGrayLevelEmphasis | GLDM-Texture | 0.007 | 0.007 | 0.000 |
| 46 | SmallDependenceLowGrayLevelEmphasis | GLDM-Texture | 0.006 | 0.064 | 0.000 |
| 47 | GrayLevelNonUniformity | GLRLM-Texture | 0.023 | 0.052 | 0.000 |
| 48 | GrayLevelNonUniformityNormalized | GLRLM-Texture | 0.008 | 0.005 | 0.000 |
| 49 | GrayLevelVariance | GLRLM-Texture | 0.007 | 0.000 | 0.000 |
| 50 | HighGrayLevelRunEmphasis | GLRLM-Texture | 0.006 | 0.094 | 0.000 |
| 51 | LongRunEmphasis | GLRLM-Texture | 0.007 | 0.651 | 0.000 |
| 52 | LongRunHighGrayLevelEmphasis | GLRLM-Texture | 0.006 | 0.602 | 0.000 |
| 53 | LongRunLowGrayLevelEmphasis | GLRLM-Texture | 0.007 | 0.494 | 0.000 |
| 54 | LowGrayLevelRunEmphasis | GLRLM-Texture | 0.006 | 0.593 | 0.000 |
| 55 | RunEntropy | GLRLM-Texture | 0.009 | 0.727 | 0.000 |
| 56 | RunLengthNonUniformity | GLRLM-Texture | 0.030 | 0.000 | 0.000 |
| 57 | RunLengthNonUniformityNormalized | GLRLM-Texture | 0.012 | 0.001 | 0.000 |
| 58 | RunVariance | GLRLM-Texture | 0.008 | 0.558 | 0.000 |
| 59 | ShortRunEmphasis | GLRLM-Texture | 0.027 | 0.000 | 0.000 |
| 60 | ShortRunHighGrayLevelEmphasis | GLRLM-Texture | 0.034 | 0.000 | 0.000 |
| 61 | ShortRunLowGrayLevelEmphasis | GLRLM-Texture | 0.007 | 0.481 | 0.000 |
| 62 | GrayLevelNonUniformity | GLSZM-Texture | 0.022 | 0.052 | 0.000 |
| 63 | GrayLevelNonUniformityNormalized | GLSZM-Texture | 0.006 | 0.005 | 0.000 |
| 64 | GrayLevelVariance | GLSZM-Texture | 0.007 | 0.000 | 0.000 |
| 65 | HighGrayLevelZoneEmphasis | GLSZM-Texture | 0.007 | 0.094 | 0.000 |
| 66 | LargeAreaEmphasis | GLSZM-Texture | 0.006 | 0.651 | 0.000 |
| 67 | LargeAreaHighGrayLevelEmphasis | GLSZM-Texture | 0.006 | 0.602 | 0.000 |
| 68 | LargeAreaLowGrayLevelEmphasis | GLSZM-Texture | 0.008 | 0.494 | 0.000 |
| 69 | LowGrayLevelZoneEmphasis | GLSZM-Texture | 0.006 | 0.593 | 0.000 |
| 70 | SizeZoneNonUniformity | GLSZM-Texture | 0.033 | 0.000 | 0.000 |
| 71 | SizeZoneNonUniformityNormalized | GLSZM-Texture | 0.008 | 0.001 | 0.000 |
| 72 | SmallAreaEmphasis | GLSZM-Texture | 0.026 | 0.000 | 0.000 |
| 73 | SmallAreaHighGrayLevelEmphasis | GLSZM-Texture | 0.031 | 0.000 | 0.000 |
| 74 | SmallAreaLowGrayLevelEmphasis | GLSZM-Texture | 0.007 | 0.481 | 0.000 |
| 75 | ZoneEntropy | GLSZM-Texture | 0.009 | 0.727 | 0.000 |
| 76 | ZoneVariance | GLSZM-Texture | 0.008 | 0.558 | 0.000 |
| 77 | Busyness | NGTDM-Texture | 0.009 | 0.004 | 0.000 |
| 78 | Coarseness | NGTDM-Texture | 0.021 | 0.113 | 0.000 |
| 79 | Contrast | NGTDM-Texture | 0.008 | 0.000 | 0.000 |
| 80 | Strength | NGTDM-Texture | 0.011 | 0.035 | 0.000 |

1. The selected features by Borutal Algorithm for building our radiomics models for ALNM task. The Settings are the same as (a).

| No. | Selected Features | Type | Impt | P(t) | P(RS) |
| --- | --- | --- | --- | --- | --- |
| 1 | Energy | Intensity | 0.04 | 0.00 | 0.00 |
| 2 | Kurtosis | Intensity | 0.04 | 0.00 | 0.00 |
| 3 | MeanAbsoluteDeviation | Intensity | 0.03 | 0.00 | 0.00 |
| 4 | Minimum | Intensity | 0.04 | 0.82 | 0.00 |
| 5 | RobustMeanAbsoluteDeviation | Intensity | 0.03 | 0.00 | 0.00 |
| 6 | Variance | Intensity | 0.04 | 0.00 | 0.00 |
| 7 | ClusterProminence | GLCM-Texture | 0.04 | 0.00 | 0.00 |
| 8 | DifferenceVariance | GLCM-Texture | 0.04 | 0.00 | 0.00 |
| 9 | SumSquares | GLCM-Texture | 0.04 | 0.00 | 0.00 |
| 10 | RunLengthNonUniformity | GLRLM-Texture | 0.06 | 0.00 | 0.00 |
| 11 | ShortRunEmphasis | GLRLM-Texture | 0.04 | 0.00 | 0.00 |
| 12 | ShortRunHighGrayLevelEmphasis | GLRLM-Texture | 0.04 | 0.00 | 0.00 |
| 13 | SizeZoneNonUniformity | GLSZM-Texture | 0.05 | 0.00 | 0.00 |
| 14 | SmallAreaEmphasis | GLSZM-Texture | 0.04 | 0.00 | 0.00 |
| 15 | SmallAreaHighGrayLevelEmphasis | GLSZM-Texture | 0.03 | 0.00 | 0.00 |
| 16 | Busyness | NGTDM-Texture | 0.03 | 0.00 | 0.00 |
| 17 | Coarseness | NGTDM-Texture | 0.04 | 0.11 | 0.00 |
| 18 | Contrast | NGTDM-Texture | 0.03 | 0.00 | 0.00 |
| 19 | Strength | NGTDM-Texture | 0.04 | 0.03 | 0.00 |

Table S3. Full P-value of metrics listed in Table 3.

|  | ALNM | | | | | | BM | | | | | |
| --- | --- | --- | --- | --- | --- | --- | --- | --- | --- | --- | --- | --- |
| Comparison pair | ACC | SE | Prec | SP | F1 | AUC | ACC | SE | Prec | SP | F1 | AUC |
| Single-BM v.s. Multi-Task | \ | \ |  | \ | \ | \ | 0.370 | 0.730 | 0.804 | 0.952 | 0.504 | 0.379 |
| Single-BM v.s. Ours | \ | \ | \ | \ | \ | \ | 0.043 | 0.137 | 0.146 | 0.242 | 0.031 | 0.127 |
| Single-ALNM v.s. Multi-Task | 0.614 | 0.064 | 0.019 | 0.810 | 0.008 | 0.597 | \ | \ | \ | \ | \ | \ |
| Single-ALNM v.s. Ours | 0.241 | 0.109 | 0.007 | 0.589 | 0.006 | 0.042 | \ | \ | \ | \ | \ | \ |
| Multi-Task v.s. Ours | 0.091 | 0.0001 | 0.0003 | 0.693 | <0.0001 | 0.009 | 0.109 | 0.654 | 0.397 | 0.444 | 0.054 | 0.300 |

Note.- t-test is adopted to assesses the significance between metrics.


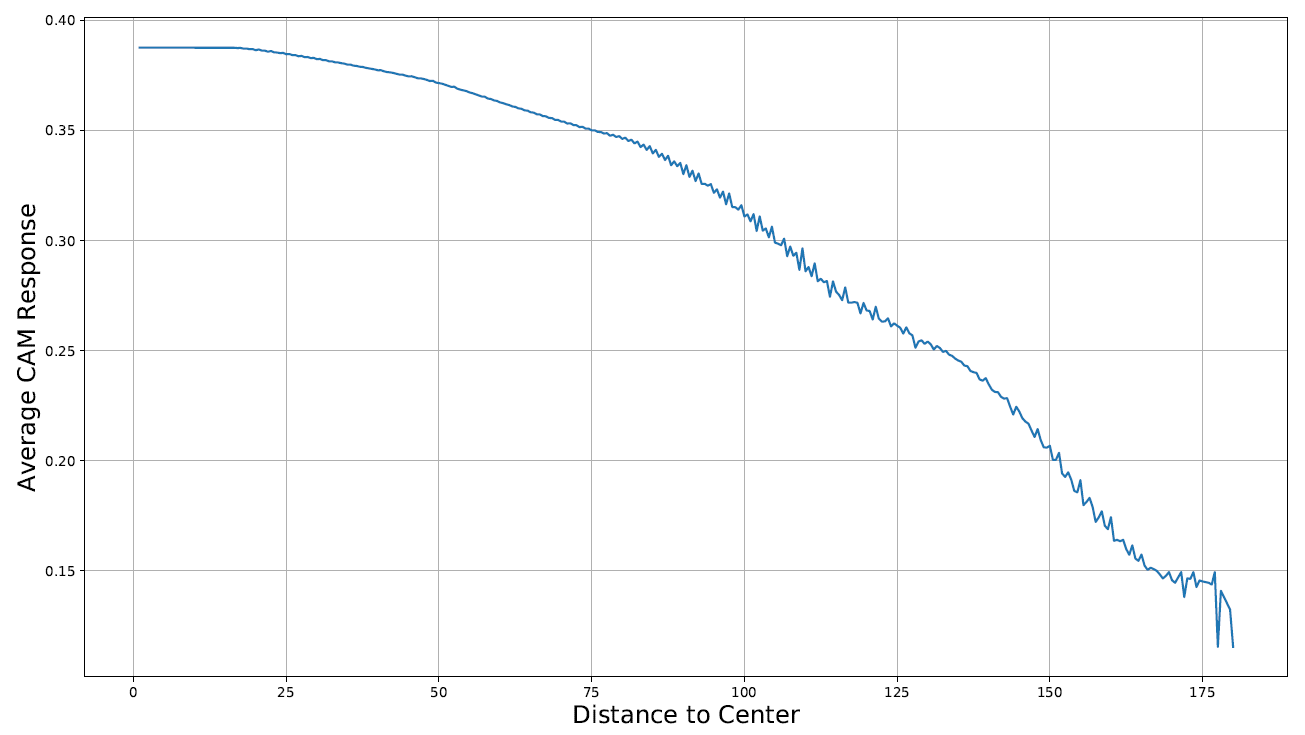


Figure S1. Average CAM Response over different distance to the canter of images on testing part. The value indicates the average significance value of the pixel for the prediction of malignant tumor and ALNM at a given distance from center pixel.


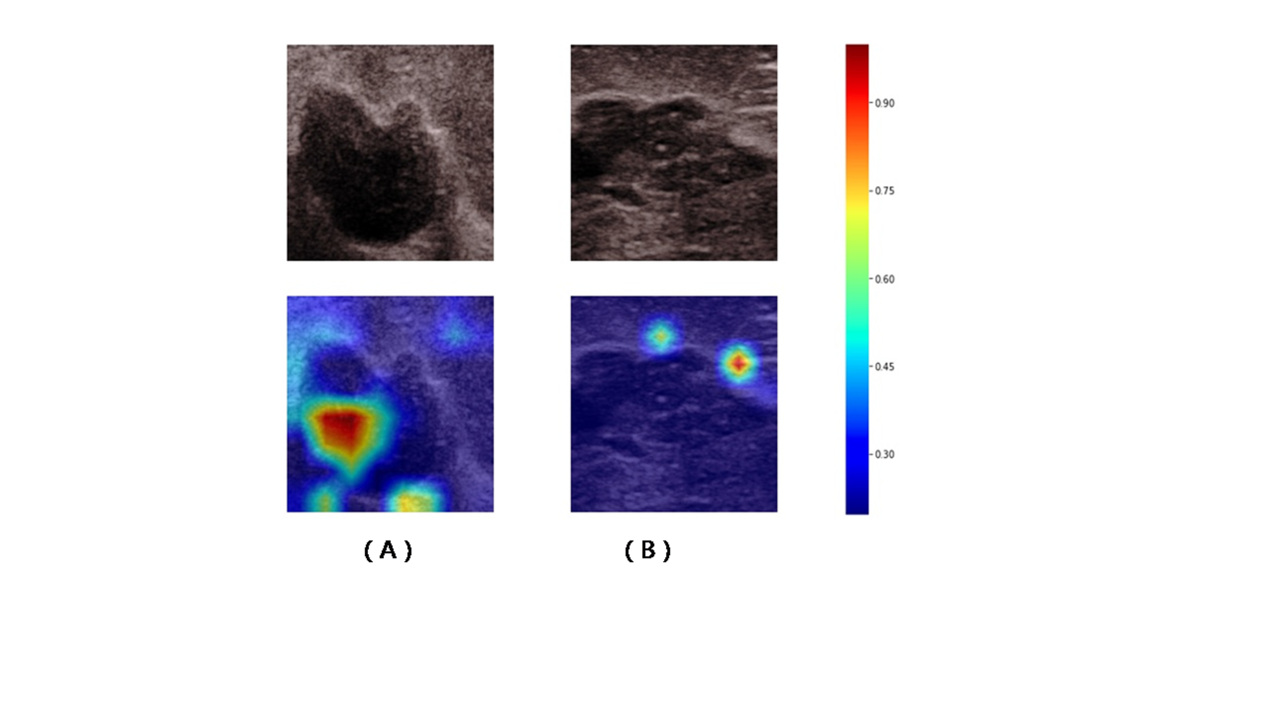


Figure S2. Failed classified cases with ground truth of 2 or 3 lymph node metastasis. (a) image and gradCAM heatmap of a 57 years old woman with invasive ductal carcinoma grade 2 and two lymph node metastasis, which is predicted as malignant andwithout lymph node metastasis. (b) image and gradCAM heatmap of a 46 years old woman with invasive ductal carcinoma grade 2-3 combined with high grade intraductal carcinoma and 3 lymph node metastasis, which is predicted as malignant and without lymph node metastasis.
